# Impact of Organo‐Functionalization on the Light‐Driven Hydrogen Evolution Reactivity of Molecular Molybdenum Sulfides

**DOI:** 10.1002/chem.71178

**Published:** 2026-05-23

**Authors:** Moritz Jahn, Luis Kammerer, Luca Schleicher, Amelie Meyer, Mariet Sibi Puthanagady, Alexander K. Mengele, Sven Rau, Stephan Kupfer, Benjamin Dietzek‐Ivanšić, Carsten Streb

**Affiliations:** ^1^ Department of Chemistry Johannes Gutenberg University Mainz Mainz Germany; ^2^ Leibniz Institute of Photonic Technology Jena Germany; ^3^ Institute For Physical Chemistry Friedrich Schiller University Jena Jena Germany; ^4^ Institute of Inorganic Chemistry I Ulm University Ulm Germany; ^5^ Leibniz Institute of Surface Engineering (IOM) Leipzig Germany

**Keywords:** hydrogen evolution, molybdenum sulfide, organo‐functionalization, photocatalysis, thiomolybdate

## Abstract

Molecular molybdenum sulfides, or thiomolybdates, are well‐established noble‐metal free molecular catalysts for the hydrogen evolution reaction (HER). To‐date, there is a knowledge‐gap regarding the impact of organo‐functionalization on reactivity, stability and heterogenization of this compound class. Here, we report the development of synthetic routes for controlled introduction of organic N‐donor ligands as a first step toward establishing structure‐property‐reactivity relationships in this new compound class. Photophysical studies combined with computational modelling are used to rationalize trends in the observed light‐driven homogeneous HER activity. This work lays the foundation to develop organo‐functionalized thiomolybdate HER catalysts suitable for covalent or supramolecular linkage to photosensitizers and for anchoring on heterogeneous supports, e.g. photocathodes.

## Graphical abstract

Organo‐functionalized molecular molybdenum sulfides were synthesized using bidentate N‐donor ligands and investigated as light‐driven homogeneous hydrogen evolution reaction (HER) catalysts. Trends observed in the HER activity of these catalysts were elucidated by combining photophysical, electrochemical and computational studies.

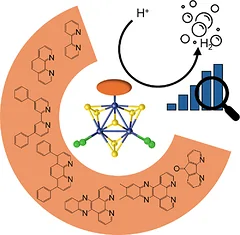

## 1 Introduction

As global energy demand rises and the energy technologies shift toward carbon neutrality, the demand for clean energy storage systems is growing rapidly. One of the most sustainable pathways to this end is the use of sunlight to produce green hydrogen, so that solar energy is converted to chemical energy, enabling a carbon‐free alternative to fossil fuels [1, 2].

Traditionally, catalysts for the hydrogen evolution reaction (HER) rely on rare and expensive noble metals such as platinum, making this approach undesirable for large‐scale deployment in industry [3]. Consequently, earth‐abundant HER catalyst alternatives have received tremendous interest [4]. Here, amorphous molybdenum sulfide catalysts have emerged as promising noble‐metal free alternative catalysts which can show comparable HER activity to Pt catalysts [5, 6, 7]. However, mechanistic studies on the molecular level of amorphous materials are inherently difficult. Therefore, molecular molybdenum sulfides, so‐called thiomolybdates, for example, the prototype [Mo_3_S_13_]^2−^ (hereafter **{Mo_3_}**) have emerged as suitable active site models for photochemical and electrochemical HER studies [7, 8, 9].

However, identifying the active sites of these molecular well‐defined catalysts is challenging as the activity is highly dependent on the catalytic conditions and multiple active species can be formed, the exact speciation and reactivity are not fully understood. Previous mechanistic studies on **{Mo_3_}** indicate that ligand exchange of the terminal disulfides has a major influence on the HER activity [10]. Also, substitution of the terminal disulfides with chloride or bromide ligands is reported and results in catalytic systems with lower HER activity compared to the native **{Mo_3_}** [10]. Mechanistic studies to‐date propose two plausible HER mechanisms which proceed either via a disulfide/sulfide mechanism [10, 11, 12] or a molybdenum hydride mechanism [6, 13]. Note that to‐date, the true nature of the active HER site in **{Mo_3_}** remains unknown, and there is experimental and theoretical evidence for both proposed mechanisms, so that in fact, both mechanisms might (co‐)exist, and the dominant mechanisms depends on the exact reaction conditions [10, 11, 12, 13].

It is noteworthy that despite the insight into the HER‐activity dependence on ligand substitution, to‐date, only few organo‐functionalized **{Mo_3_}** derivatives have been reported [6, 14, 15, 16, 17, 18] and HER studies on these systems are exceedingly rare [19, 20]. In 2019 Schmehl and colleagues [19] introduced dithiocarbamate ligands to the [Mo_3_S_7_]^4+^ core, providing insights into the HER performance of the catalyst in the absence of terminal disulfide ligands [19]. The group reported structural changes of the catalyst under irradiation, involving the conversion of bridging disulfides to sulfides [19]. Recently Lin and coworkers [20] reported 2‐mercaptobenzothiazole substituted **{Mo_3_}**‐based catalysts with high acid‐base stability, and reported initial photocatalytic HER data [20].

**FIGURE 1:**
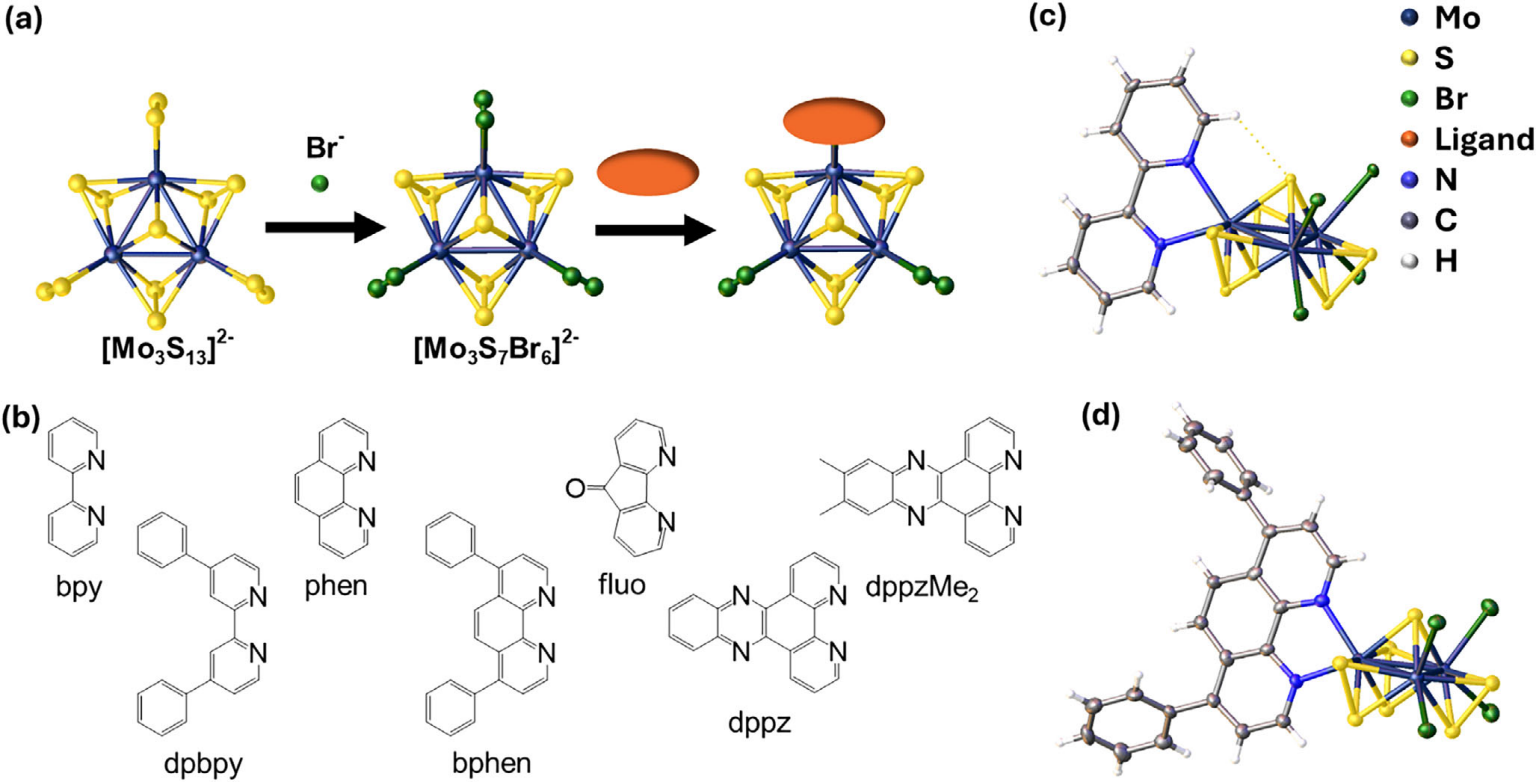
(a) Synthesis scheme of the organo‐functionalization of thiomolybdate clusters and (b) illustration of the N‐donor ligands employed, that is, 2,2′‐bipyridine (bpy); 1,10‐phenanthroline (phen); 4,4'‐diphenyl‐2,2'‐bipyridine (dpbpy); bathophenanthroline (bphen); dipyrido[3,2‐a:2',3'‐c]phenazine (dppz); 7,8‐dimethyldipyridophenazine (dppzMe_2_); 4,5‐diazafluoren‐9‐one (fluo). (c) Crystal structure of **{Mo_3_Br}bpy**, C (grey), H (white), Br (green), Mo (dark blue), N (blue), S (yellow), probability ellipsoids at 50%. Solvent molecules and TEABr were removed for clarity. (d) Crystal structure of **{Mo_3_Br}bphen**, C (grey), H (white), Br (green), Mo (dark blue), N (blue), S (yellow), probability ellipsoids at 50%. Solvent molecules and TEABr were removed for clarity.

Here, we build on these pioneering studies and report synthetic access and mechanistic insights for a series of organo‐functionalized **{Mo_3_}** derivatives with the general formula TEA[Mo_3_S_7_Br_4_L]Br (TEA = tetraethylammonium, L = ligand). As ligand L, we used chelating N‐donor ligands. The more flexible 2,2′‐bipyridine type was employed to investigate the most optimal coordination geometry at the molybdenum center allowed for by the free rotation around the C2─C2’ bond. Specifically 2,2′‐bipyridine (bpy); 4,4'‐diphenyl‐2,2'‐bipyridine (dpbpy) were employed. The more rigid and therefore demanding 1,10‐phenanthroline type was investigated by using 1,10‐phenanthroline (phen); bathophenanthroline (bphen); dipyrido[3,2‐a:2',3'‐c]phenazine (dppz); 7,8‐dimethyldipyridophenazine (dppzMe_2_). By utilizing the 4,5‐diazafluoren‐9‐one (fluo) similar rigidity of the ligand as observed with phenanthroline can be anticipated, however the relative position of the N‐donor atoms is significantly altered in comparison to the former. All ligands are shown in Figure 1b. We compared the light‐driven HER performance of these compounds and evaluated their activity with electrochemical, photophysical and theoretical studies, linking their HER activity to their reduction potential, catalyst‐photosensitizer interactions and frontier orbital energies.

## 2 Results and Discussion

### 2.1 Synthesis and Characterization

**{Mo_3_}** was synthesized based on a literature‐reported procedure [10]. Next, **{Mo_3_}** was brominated using hydrobromic acid as reported by Fedin and colleagues [21] (resulting in [Mo_3_S_7_Br_6_]^2−^, **{Mo_3_Br}**), followed by substitution of the bromide ligands with the respective organic ligand (Figure 1a). The reaction of **{Mo_3_Br}** with the respective ligands was performed similar to a literature‐reported method [17] and adapted for each ligand. This gave the compounds TEA[Mo_3_S_7_Br_4_L]Br (hereafter **{Mo_3_Br}L**) (L = N‐donor ligand, for details see Figure 1a). All clusters were characterized by ^1^H‐NMR spectroscopy, IR spectroscopy, UV‐Vis spectroscopy, elemental analysis (EA) and where possible by single crystal x‐ray diffraction (SC‐XRD). Single crystals suitable for SC‐XRD were obtained for **{Mo_3_Br}bpy** and **{Mo_3_Br}bphen** (Figure 1c,d; see Supporting Information for crystallographic details) [22]. For both **{Mo_3_Br}bpy** and **{Mo_3_Br}bphen** one ligand is coordinated to a molybdenum center, resulting in a charge‐neutral cluster. Additionally, a bromide ion is bound near the cluster via the bridging disulfides, which is charge‐balanced by a TEA cation in the lattice. This unusual phenomenon has been observed previously [23] and assigned to weak disulfide‐bromide interactions. The crystal structure of **{Mo_3_Br}bpy** was previously reported by Nandi and colleagues [16]. As shown in Figure 1c,d, the ligands show virtually no structural distortion. ^1^H‐NMR spectroscopy of all compounds was performed in DMSO‐d_6_. The functionalized clusters were compared to the free ligand, all showing characteristic downfield shifts as well as splitting of the signals due to reduced symmetry of resulting species (for complete data, see Supporting Information). As expected, the strongest signal splitting is observed for the protons in proximity of the thiomolybdate cluster, indicating the spatial proximity of ligand and cluster also in solution.

### 2.2 Light‐Driven HER Studies

**FIGURE 2:**
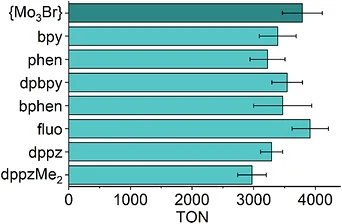
TONs of all catalysts studied. Functionalized catalysts are abbreviated with the respective ligand. Conditions: catalyst (0.3 µM), photosensitizer [Ru(bpy)_3_](PF_6_)_2_ (20 µM), SED: ascorbic acid (0.25 M) solvent: MeOH containing 5 vol‐% DMF. LED irradiation, *λ* = 467 nm, irradiation time 6 h.

Light‐driven HER studies were performed for all organo‐functionalized catalysts and compared to **{Mo_3_Br}** as a reference. Measurements were performed using samples containing the respective catalyst (0.3 µM), [Ru(bpy)_3_](PF_6_)_2_ (20 µM) as photosensitizer and ascorbic acid (0.25 M) as sacrificial electron donor (SED) in MeOH containing 5 vol‐% N,N‐dimethyl formamide (DMF). Ascorbic acid was established previously as suitable SED for comparable catalytic systems using **{Mo_3_}** as catalyst [24]. Additionally ascorbic acid is known for tolerating acidic conditions, whereas basic conditions are usually necessary for other commonly used amine‐based SEDs to prevent protonation, which severely limits their electron‐donating capabilities [25]. The samples were irradiated at *λ* = 467 nm (LED) for 6 h, for details see Supporting Information. Hydrogen production was quantified via head‐space gas chromatography (GC) and all data shown were recorded multiple times to obtain meaningful averages and standard deviations (for details see, Table S2). The resulting turnover numbers (TONs) are shown in Figure 2. The native species **{Mo_3_Br}** shows a TON of ca. 3790 and for all ligand substitutions studied, we observe only modest drops in TON, highlighting that ligand substitution is possible while maintaining the overall light‐driven HER activity of the species. Notably, for **{Mo_3_Br}fluo**, we observe slightly higher TONs (ca. 3920) compared to the native **{Mo_3_Br}** species. Reference experiments performed without [Ru(bpy)_3_]^2+^ show strongly reduced catalytic activity, with TONs <50 (see Table S3), emphasizing the need of a photosensitizer for effective visible light HER. When compared to other thiomolybdate HER systems we note that the systems reported show comparable reactivity with other organo‐functionalized species (e.g., TON ca. 300 for dithiocarbamate functionalized systems [19]), however, we also note that these systems do not achieve the activity reported for the native [Mo_3_S_13_]^2−^ (TONs > 23,000) [10].

To gain quantitative insights into the energy necessary to produce hydrogen in our system, we determined the photonic efficiency (PE, details see Supporting Information). The PE reaches from 2.87 × 10^−3^ for **{Mo_3_Br}dppzMe_2_** to 3.78 × 10^−3^ for **{Mo_3_Br}fluo**. Thus, the PE is in the same order of magnitude as for previously reported data for **{Mo_3_}** [24].

In order to assess the stability of the catalysts, we investigated structural changes of the catalyst in solution by ^1^H‐NMR before and after irradiation (see Figures S24–S30). Since characterization of the catalyst under catalytic conditions is very challenging, we used a simplified system, enabling suitable concentrations for NMR spectroscopy and avoiding interfering peaks from other compounds. Therefore, 10 mM solutions of the respective catalysts were prepared in DMSO‐d_6_ and irradiated for 6 h using a 467 nm LED. The data show that the major NMR signals associated with the intact species are present after irradiation, while in some cases, some minor additional signals are observed. While the exact nature of these signals is currently unknown, the data demonstrate the overall stability and integrity of the systems, and no ligand exchange (i.e., no signals for “free” ligands) is observed in our studies.

### 2.3 Electrochemistry and Computational Modelling

**FIGURE 3:**
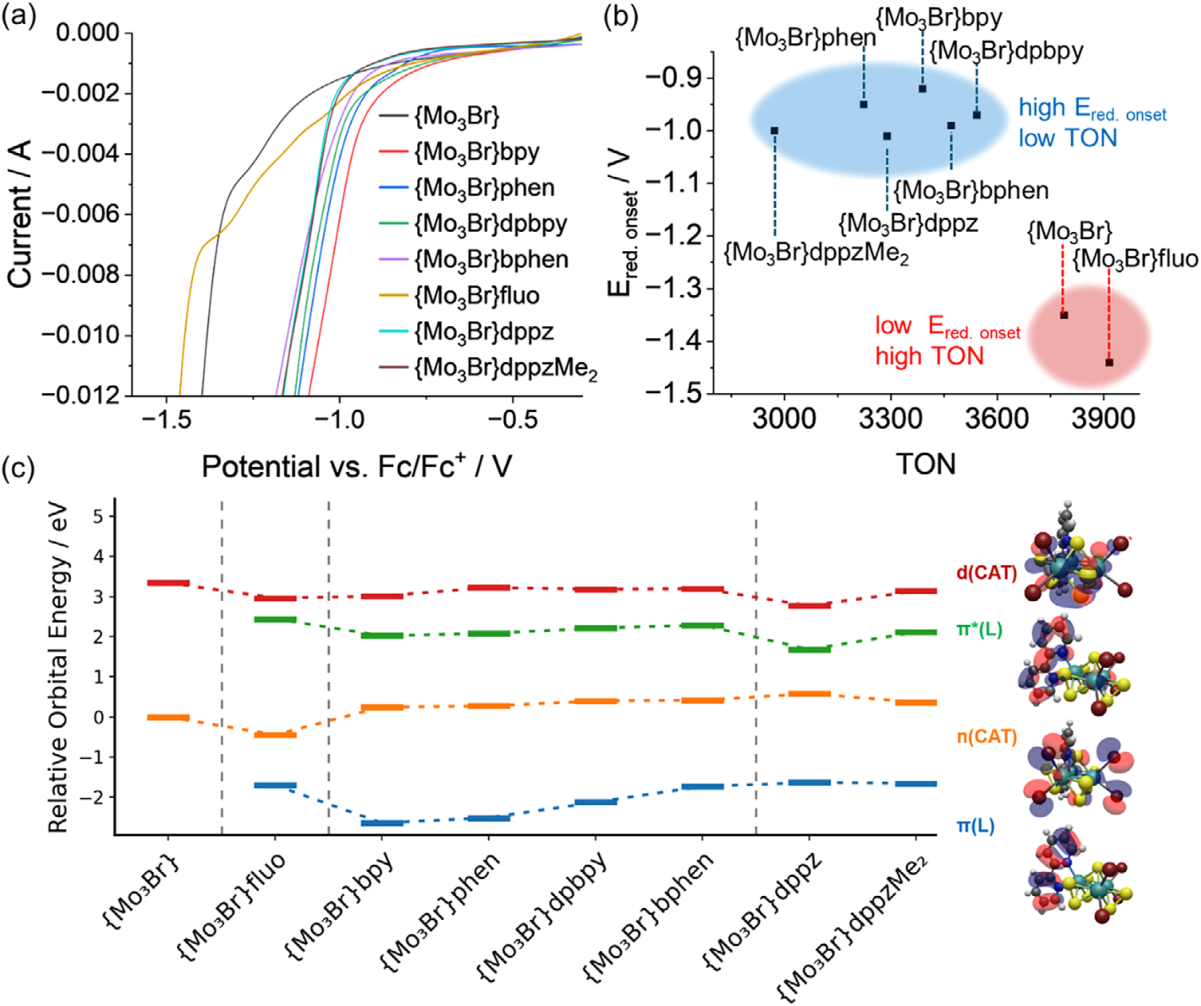
(a) Linear sweep voltammograms of all catalysts (1 mmol/L) in de‐aerated DMF containing 0.1 mM nBu_4_NPF_6_; scan rate 100 mV/s; internal reference: Fc^+/0^. (b) Relationship between the reduction onset potential E_red. onset_ and the observed turnover number TON for the catalysts studied. (c) DFT‐calculated frontier orbital energy levels of **{Mo_3_Br}** and ligand‐substituted derivatives. Energies of ligand‐centered π(L)/π*(L) and metal‐centered d(CAT)/n(CAT) orbitals are shown relative to the d(CAT) orbital of **{Mo_3_Br}** (set to 0 eV). Vertical dashed lines indicate ligand groupings; orbitals are visualized for **{Mo_3_Br}bpy**.

To explore possible differences in electron transfer behavior, we explored the electrochemistry of the catalysts. In order to access the onset potential for cluster reduction, linear sweep voltammetry (LSV) was performed (Figure 3). All compounds except **{Mo_3_Br}** and **{Mo_3_Br}fluo** show a clear first reduction event between −0.92 V and −1.00 V vs the Fc^+^ /Fc° couple. The reduction event for **{Mo_3_Br}** and **{Mo_3_Br}fluo** are both cathodically shifted to more negative potentials (−1.35 V and −1.44 V, respectively) and show a more complex reduction pattern, which could indicate possible structural changes or more complex electron‐transfer processes upon reduction. The compounds with significantly more negative reduction potential also show higher average HER activity, but no clear trend between reduction onset potential and HER is observable, indicating other competing effects. These could include supramolecular interaction of photosensitizer and catalyst, aggregate formation, or photoinduced catalyst / photosensitizer degradation.

Furthermore, density functional theory (DFT) calculations (see Supporting Information for computational setup) were performed to investigate the energetics as well as the electronic characters of the frontier molecular orbitals along the series of thiomolybdate clusters. In particular, these quantum chemical simulations aimed to assess the energy levels of the lowest virtual orbitals localized on the **{Mo_3_Br}** cluster and the introduced aromatic ligand, and thus to elucidate the molecular moieties which are associated with the first reduction event (Figure 3c); the calculated orbital energies are shown in the Supporting Information (Table S8). The lowest‐unoccupied molecular orbitals (LUMOs) range from 1.68 eV for **{Mo_3_Br}dppz** to 3.34 eV for **{Mo_3_Br}** relative to the d(CAT) orbital of **{Mo_3_Br}** (set to 0 eV). Upon introduction of the organic ligand all thiomolybdate clusters localize the LUMO on the organic ligands. Notably, such ligand modification also affects the energy levels of the **{Mo_3_Br}**‐centered highest‐occupied molecular orbital (HOMO)—which is mainly given by the lone‐pairs of the bromide ligands—as well as of the respective cluster‐based LUMOs. Thus, introduction of these ligands allows us to tailor the electrochemical properties and in consequence the catalytic performance (see Table S7). Since the active H‐binding site is expected to be on the [Mo_3_S_7_]^4+^ core of the catalyst [6], a further stabilization of the cluster's lowest virtual orbital toward or even below the π(L)* orbital might be beneficial for HER activity. The LUMOs of **{Mo_3_Br}fluo**, **{Mo_3_Br}dppz** and **{Mo_3_Br}dppzMe_2_** are mainly ligand‐centered with only little electron density on the cluster part, while the LUMOs for all other catalysts are distributed over the whole catalyst. It is noteworthy that in the case of **{Mo_3_Br}fluo** the π(L)* orbital is the closest in energy to the d(CAT) orbital. This observation may account for the enhanced catalytic performance of **{Mo_3_Br}fluo**. As the π‐system of the ligand extends, reduction at the ligand becomes progressively more favorable, thereby competing with and disfavoring reduction at the cluster. In the case of **{Mo_3_Br}fluo**, cluster‐centered reduction is therefore least impeded. Hence, electron‐withdrawing ligands featuring comparatively small π‐systems, like fluo, may have a positive effect on catalysis, whereas electron‐rich ligands with extended π‐systems, such as dppz or dppzMe_2_, may lead to reduced catalytic activity. Notably, this initial computational modelling merely allows us to assess the respective electrochemical properties along the series of molecular molybdenum sulfide clusters, while future joint synthetic‐spectroscopic‐theoretical investigations will focus on an in‐depth evaluation of the subsequent reaction pathways associated with HER activity.

### 2.4 Stern–Volmer Analysis

**FIGURE 4:**
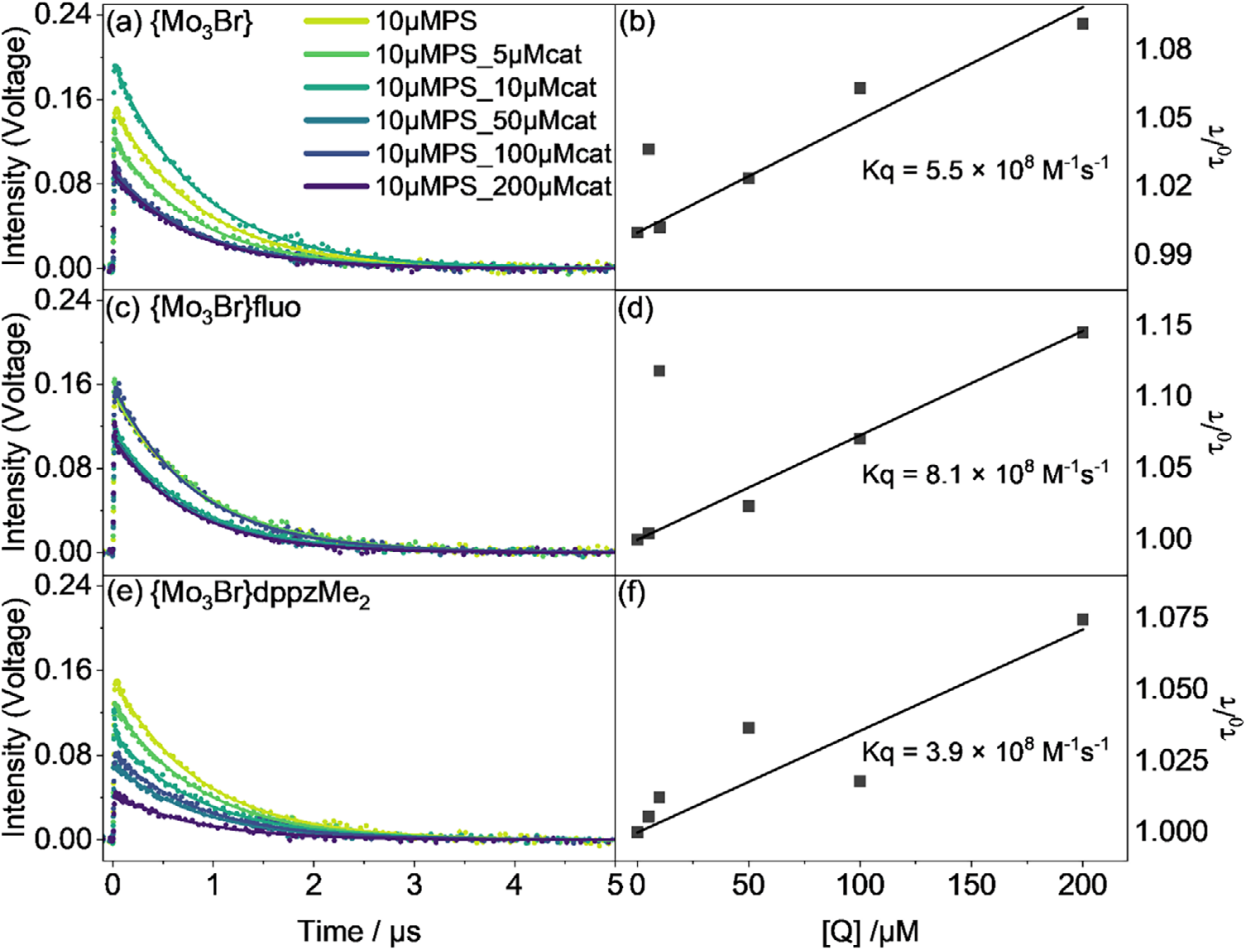
Decay kinetics (610 nm) of photosensitiser in presence of different concentration of the quencher at *λ*_ex_ = 470 nm (a) **{Mo_3_Br}** (c) **{Mo_3_Br}fluo** (e) **{Mo_3_Br}dppzMe_2_**. Stern–Volmer plots showing quenching efficiency of the quencher (b) **{Mo_3_Br}**, (d) **{Mo_3_Br}fluo,** and (f) **{Mo_3_Br}dppzMe_2_**.

Next, we were interested to study solution interactions between the photosensitizer and the respective catalysts, which is relevant to understand catalytic performance, particularly since supramolecular π‐stacking interactions can control intermolecular / supramolecular charge‐transfer in related systems [26]. Here, we used emission quenching studies and Stern–Volmer analyses to gain insights into the solution behavior and interactions between the photosensitizer, [Ru(bpy)_3_]^2+^ and the three model catalysts **{Mo_3_Br}** (reference), **{Mo_3_Br}fluo** (highest TONs) and **{Mo_3_Br}dppzMe_2_** (lowest TONs). Figure 4 summarises the results from emission quenching experiments where decay kinetics of the photosensitizer at 610 nm in the presence of three catalysts (a, c, e) shows a clear quenching trend in emission intensity. The decrease in the excited state lifetime of the photosensitizer (τ) with increase of quencher concentration [Q] suggests a dynamic quenching mechanism due to collisional interactions between excited state photosensitiser and catalyst and implies an oxidative quenching pathway under the given conditions. For all three systems, the bimolecular quenching rate constants are on the order of 10^8^ M^−1^ s^−1^ (b, d, f), which confirms a moderate level of quenching with **{Mo_3_Br}fluo** showing a slightly higher bimolecular quenching rate (*K*_q_ = 8.1 × 10^8^ M^−1^ s^−1^) in comparison to **{Mo_3_Br}dppzMe_2_** which shows the lowest (*K*_q_ = 3.9 × 10^8^ M^−1^ s^−1^) while **{Mo_3_Br}** (reference) lies in between these two extremes (*K*_q_ = 5.5 × 10^8^ M^−1^ s^−1^). This is in line with the observed light‐induced catalytic HER performance of the three systems, thereby, suggesting that organo‐functionalization of thiomolybdates can be used to control solution‐interactions between a photosensitizer and the respective catalyst.

## 3 Conclusion

In summary, we report the first systematic synthetic access to thiomolybdate HER catalysts organo‐functionalized with aromatic N‐donor ligands. Catalytic studies show that the catalysts retain their HER activity after ligand exchange, thereby opening new paths for the development of organic‐inorganic hybrid HER catalysts. Initial mechanistic studies using photophysical analyses and theoretical computations provide first insights into the factors which affect HER‐activity and point to areas which require further study to rationalize the tuning scope for this new compound class. In future, we expect that these species can be used for the design of covalently or non‐covalently linked photosensitizer‐catalyst dyads, as well as for the cluster anchoring on heterogeneous substrates, for example, photoelectrodes.

## Conflicts of Interest

The authors declare no conflicts of interest.

## Supporting information



The authors have cited additional references within the Supporting Information [27, 28, 29, 30, 31, 32, 33, 34, 35, 36, 37].

Supporting file: chem71178‐sup‐0002‐DataFile.cif.

## Data Availability

The data that support the findings of this study are openly available in Zenodo at https://zenodo.org, reference number 18712717.

## References

[1] IEA , “ Net Zero by 2050‐ A Roadmap for the Global Energy Sector ”, can be found under, 2021 (accessed: 12.02.2026) https://www.iea.org/reports/net‐zero‐by‐2050.

[2] R. Eisenberg and D. G. Nocera , “Preface: Overview of the Forum on Solar and Renewable Energy,” Inorganic Chemistry 44 (2005): 6799–6801. 16180837 10.1021/ic058006i

[3] S. Cao , L. Piao , and X. Chen , “Emerging Photocatalysts for Hydrogen Evolution,” Trends in Chemistry 2 (2020): 57–70.

[4] Š. Kment , A. Bakandritsos , I. Tantis , et al., “Single Atom Catalysts Based on Earth‐Abundant Metals for Energy‐Related Applications,” Chemical Reviews 124 (2024): 11767–11847. 38967551 10.1021/acs.chemrev.4c00155 PMC11565580

[5] C. G. Morales‐Guio and X. Hu , “Amorphous Molybdenum Sulfides as Hydrogen Evolution Catalysts,” Accounts of Chemical Research 47 (2014): 2671–2681. 25065612 10.1021/ar5002022

[6] S. Batool , M. Langer , S. N. Myakala , et al., “Thiomolybdate Clusters: From Homogeneous Catalysis to Heterogenization and Active Sites,” Advanced Materials 36 (2024): 2305730. 37899494 10.1002/adma.202305730 PMC11475511

[7] M. L. Grutza , A. Rajagopal , C. Streb , and P. Kurz , “Hydrogen Evolution Catalysis by Molybdenum Sulfides (MoS_x_): Are Thiomolybdate Clusters Like [Mo_3_S_13_]^2−^ Suitable Active Site Models?,” Sustainable Energy Fuels 2 (2018): 1893–1904.

[8] J. Kibsgaard , T. F. Jaramillo , and F. Besenbacher , “Building an Appropriate Active‐site Motif Into a Hydrogen‐Evolution Catalyst With Thiomolybdate [Mo_3_S_13_]^2−^ Clusters,” Nature Chemistry 6 (2014): 248–253. 10.1038/nchem.1853 24557141

[9] M. Heiland , R. De , S. Rau , B. Dietzek‐Ivansic , and C. Streb , “Not That Innocent—Ammonium Ions Boost Homogeneous Light‐driven Hydrogen Evolution,” Chemical Communications 58 (2022): 4603–4606. 35311842 10.1039/d2cc00339b

[10] M. Dave , A. Rajagopal , M. Damm‐Ruttensperger , et al., “Understanding Homogeneous Hydrogen Evolution Reactivity and Deactivation Pathways of Molecular Molybdenum Sulfide Catalysts,” Sustainable Energy Fuels 2 (2018): 1020–1026.

[11] A. Baloglou , M. Ončák , M. L. Grutza , C. Van Der Linde , P. Kurz , and M. K. Beyer , “[Mo_3_S_13_]^2−^, [HMo_3_S_13_]^−^, and [H_3_Mo_3_S_13_]^+^ as Model Systems of a Promising Hydrogen Evolution Catalyst,” Journal of Physical Chemistry 123 (2019): 8177–8186. 10.1021/acs.jpcc.8b08324 PMC6453024 30984322

[12] C. H. Lee , S. Lee , Y. K. Lee , et al., “Understanding the Origin of Formation and Active Sites for Thiomolybdate [Mo_3_S_13_]^2−^ Clusters as Hydrogen Evolution Catalyst Through the Selective Control of Sulfur Atoms,” ACS Cataysis 8 (2018): 5221–5227.

[13] P. D. Tran , T. V. Tran , M. Orio , et al., “Coordination Polymer Structure and Revisited Hydrogen Evolution Catalytic Mechanism for Amorphous Molybdenum Sulfide,” Nature Materials 15 (2016): 640–646. 26974410 10.1038/nmat4588 PMC5495159

[14] D. Recatalá , R. Llusar , F. Galindo , K. A. Brylev , and A. L. Gushchin , “Heteroleptic Phenanthroline Complexes of Trinuclear Molybdenum Clusters With Luminescent Properties,” European Journal of Inorganic Chemistry 2015 (2015): 1877–1885.

[15] D. Recatalá , R. Llusar , A. Barlow , et al., “Synthesis and Optical Power Limiting Properties of Heteroleptic Mo_3_S_7_ Clusters,” Dalton Transactions 44 (2015): 13163–13172. 26110541 10.1039/c5dt01244a

[16] G. Nandi , S. Sarkar , B. S. Reddy , T. Y. Kim , and K. M. Tripathi , “Heteroleptic Bipyridine Complex: Synthesis, Spectral, and Structural Analyses, and Interconversion of Its {Mo_3_S_7_} Core to {Mo_3_S_4_} Core,” Journal of Molecular Structure 1234 (2021): 130138.

[17] A. Gushchin , R. Llusar , D. Recatalá , and P. Abramov , “First Heteroleptic Mo_3_S_7_ Clusters Containing Non‐Innocent Phenanthroline Ligands,” Russian Journal of Coordination Chemistry 38 (2012): 173–177.

[18] V. P. Fedin , Y. V. Mironov , A. V. Virovets , N. V. Podberezskaya , and V. Y. Fedorov , “Synthesis and X‐ray Structure of the Triangular Cluster (Et_4_N){[Mo_3_(μ_3_‐S)(μ_2_‐S_2_)_3_(NH_2_Ph)_3_Br_3_]Br}Br,” Polyhedron 11 (1992): 2083–2088.

[19] P. R. Fontenot , B. Shan , B. Wang , et al., “Photocatalytic H2‐Evolution by Homogeneous Molybdenum Sulfide Clusters Supported by Dithiocarbamate Ligands,” Inorganic Chemistry 58 (2019): 16458–16474. 31790221 10.1021/acs.inorgchem.9b02252

[20] H. L. Zheng , J. Q. Zhao , J. Zhang , and Q. Lin , “Acid–Base Resistant Ligand‐Modified Molybdenum–Sulfur Clusters With Enhanced Photocatalytic Activity Towards Hydrogen Evolution,” Journal of Materials Chemistry A 10 (2022): 7138–7145.

[21] V. P. Fedin , M. N. Sokolov , Y. V. Mironov , B. A. Kolesov , S. V. Tkachev , and V. Y. Fedorov , “Triangular Thiocomplexes of Molybdenum: Reactions With Halogens, Hydrohalogen Acids and Phosphines,” Inorganica Chimica Acta 167 (1990): 39–45.

[22] Deposition numbers 874761 (for {Mo3Br}bpy), and 2530461 (for {Mo3Br}bphen) contain the supplementary crystallographic data for this paper. These data are provided free of charge by the joint Cambridge Crystallographic Data Centre and Fachinformationszentrum Karlsruhe Access Structures service.

[23] P. Klingelhöfer , U. Müller , C. Friebel , and J. Pebler , “Thiochloroanionen von Molybdän(IV). Die Kristallstruktur von (NEt_4_)_3_[Mo_3_(μ_3_‐S)(μ‐S_2_)_3_Cl_6_]Cl · CH_2_Cl_2_ Kristallstruktur, EPR‐Spektrum und Magnetische Eigenschaften von (NEt_4_)_2_[Mo_2_(μ‐S_2_)(μ‐Cl)_2_Cl_6_],” Zeitschrift für anorganische und allgemeine Chemie 543 (1986): 22–34.

[24] S. H. Kolbinger , L. Haxha , P. C. Schröder , et al., “Fewer Photons, More Hydrogen: Effects of Dynamic Irradiation on Light‐driven Hydrogen Evolution by Thiomolybdate Catalysts,” Sustainable Energy Fuels 10 (2026): 1313–1320.

[25] Y. Pellegrin and F. Odobel , “Sacrificial Electron Donor Reagents for Solar Fuel Production Les Donneurs d′ electron Sacrificiels Pour la Production de Combustible Solaire,” Comptes rendus—Chimie 20 (2017): 283–295.

[26] M. G. G. Pfeffer , C. Pehlken , R. Staehle , D. Sorsche , C. Streb , and S. Rau , “Supramolecular Activation of a Molecular Photocatalyst,” Dalton Transactions 43 (2014): 13307–13315. 25060863 10.1039/c4dt00761a

[27] F. Neese , “Software Update: The ORCA Program System—Version 6.0,” Wiley Interdisciplinary Reviews: Computational Molecular Science 15 (2025): e70019.

[28] A. D. Becke , “Density‐functional Thermochemistry. III. The Role of Exact Exchange,” Journal of Chemical Physics 98 (1993): 5648–5652.

[29] F. Weigend and R. Ahlrichs , “Balanced Basis Sets of Split Valence, Triple Zeta Valence and Quadruple Zeta Valence Quality for H to Rn: Design and Assessment of Accuracy,” Physical Chemistry Chemical Physics 7 (2005): 3297–3305. 16240044 10.1039/b508541a

[30] S. Grimme , S. Ehrlich , and L. Goerigk , “Effect of the Damping Function in Dispersion Corrected Density Functional Theory,” Journal of Computational Chemistry 32 (2011): 1456–1465. 21370243 10.1002/jcc.21759

[31] S. Grimme , J. Antony , S. Ehrlich , and H. Krieg , “A Consistent and Accurate Ab Initio Parametrization of Density Functional Dispersion Correction (DFT‐D) for the 94 Elements H‐Pu,” Journal of Chemical Physics 132 (2010): 154104. 20423165 10.1063/1.3382344

[32] D. Andrae , U. Häußermann , M. Dolg , H. Stoll , and H. Preuß , “Energy‐adjusted Ab Initio Pseudopotentials for the Second and Third Row Transition Elements,” Theoretica chimica acta 77 (1990): 123–141.

[33] M. D. Hanwell , D. E. Curtis , D. C. Lonie , T. Vandermeerschd , E. Zurek , and G. R. Hutchison , “Avogadro: An Advanced Semantic Chemical Editor, Visualization, and Analysis Platform,” Journal of Cheminformatics 4 (2012): 17. 22889332 10.1186/1758-2946-4-17 PMC3542060

[34] Avogadro: An Open‐Source Molecular Builder and Visualization Tool. Version 1.102.3, http://avogadro.cc/.

[35] M. Stephenson , C. Reichardt , M. Pinto , et al., “Ru(II) Dyads Derived From 2‐(1‐pyrenyl)‐1H‐imidazo[4,5‐f][1,10]Phenanthroline: Versatile Photosensitizers for Photodynamic Applications,” Journal of Physical Chemistry A 118 (2014): 10507–10521. 24927113 10.1021/jp504330s

[36] W. Travis , C. E. Knapp , C. N. Savory , et al., “Hybrid Organic‐Inorganic Coordination Complexes as Tunable Optical Response Materials,” Inorganic Chemistry 55 (2016): 3393–3400. 26974692 10.1021/acs.inorgchem.5b02749

[37] A. Kleineweischede and J. Mattay , “Synthesis of Amino‐ and Bis(bromomethyl)‐Substitued Bi‐ and TetradentateN‐Heteroaromatic Ligands: Building Blocks for Pyrazino‐Functionalized Fullerene Dyads,” European Journal of Organic Chemistry 2006 (2006): 947–957.

