# Supplementary material for: Impact of Organo‐Functionalization on the Light‐Driven Hydrogen Evolution Reactivity of Molecular Molybdenum Sulfides
Source: Chemistry. 2026 May 23;32(33):e71178. doi: 10.1002/chem.71178 (PMC13532132; doi:10.1002/chem.71178)
Supplement: Supplementary file 1 [file CHEM-32-e71178-s002.docx]

**Supporting Information for**

Impact of Organo-Functionalization on the Light-driven Hydrogen Evolution Reactivity of Molecular Molybdenum Sulfides

Moritz Jahn^a^, Luis Kammerer^a^, Luca Schleicher^a^, Amelie Meyer^a^, Mariet Sibi Puthanagady^b,d^, Alexander K. Mengele^c^, Sven Rau^c^, Stephan Kupfer^d^, Benjamin Dietzek-Ivanšić^b,d,e^*, Carsten Streb^a^*

^a^ Department of Chemistry, Johannes Gutenberg University Mainz, Duesbergweg 10-14, 55128 Mainz, Germany

^b^ Leibniz Institute of Photonic Technology, Albert Einstein-Strasse 9, 07745 Jena, Germany

^c^ Institute of Inorganic Chemistry I, Ulm University, Albert-Einstein-Allee 11, 89081 Ulm, Germany

^d^ Institute for Physical Chemistry, Friedrich Schiller University Jena, Lessingstrasse 4, 07743 Jena, Germany

^e^ Leibniz Institute of Surface Engineering (IOM), Permoserstrasse 15, 04318 Leipzig, Germany

[1.	Instrumentation	2](#_Toc227567242)

[2.	Experimental Data	3](#_Toc227567243)

[2.1.	Synthesis of organic functionalized thiomolybdates	3](#_Toc227567244)

[2.2.	Photocatalytic HER Measurements	5](#_Toc227567245)

[HER Catalysis	5](#_Toc227567246)

[Catalytic reference measurements without photosensitizer	5](#_Toc227567247)

[Determination of photonic efficiency	6](#_Toc227567248)

[2.3.	Characterization of compounds	7](#_Toc227567249)

[^1^H-NMR spectra	7](#_Toc227567250)

[CHN Elemental Analysis	9](#_Toc227567251)

[ATR-FT-IR spectra	10](#_Toc227567252)

[UV-Vis spectra	14](#_Toc227567253)

[Single crystal XRD data	18](#_Toc227567254)

[2.4.	Computational Data	20](#_Toc227567255)

[Visualization of frontier orbitals of all catalysts	21](#_Toc227567256)

[2.5 Catalyst stability	23](#_Toc227567257)

[3.	References	27](#_Toc227567258)

# 1. Instrumentation

**Attenuated total Reflectance-Fourier transform Infrared Spectroscopy** (ATR-FTIR) was carried out on a Bruker Alpha II FTIR spectrophotometer containing a Diamond crystal ATR unit. Signals are presented as wave numbers in cm^-1^. The data were recorded with 24 scans at a resolution of 4 cm^-1^. All spectra were background-corrected within the Bruker OPUS 8.1 program suite.

**^1^H-NMR spectroscopy:** ^1^H spectra were recorded on Bruker AVANCE NEO 400 MHz Spectrometer equipped with 5mm inverse probe head operating at 400.13 MHz ^1^H frequency at ambient temperature conditions. Chemical shifts value (δ) for are given in part per million (ppm) using residual solvent protons (δ_H_ = 2.50 ppm for DMSO-d_6_).

**Elemental analysis:** The C, H, N, S analyses of all compounds were carried out in duplicates or triplicates on an Elementar Vario Micro Cube elemental analyzer equipped with a thermal conductivity detector (TCD), suitable for determining contents of C, H, and N, as well as an infrared detector (NDIR), suitable for detection of S. The individual C, H, N, S values were corrected using a standard sample of sulfanilamide (EA Grade, Elementar Analysensysteme GmbH). The mean C, H, N, S values are given for each compound.

**Single-crystal X-ray diffraction (sc-XRD)** was measured on a Bruker APEX-II CCD Single-crystal X-ray diffractometer equipped with a graphite monochromator using Mo Kα radiation (wavelength λ(Mo Kα) = 0.71073 Å).

**UV/Vis/NIR spectroscopy** was performed on a Cary 3500 UV/Vis/NIR spectrophotometer equipped with a Xenon flash lamp (250 Hz). Measurements were performed in quartz glass cuvettes (d = 10.0 mm).

**Gas chromatography** was performed on a Shimadzu Nexis GC-2030 equipped with a dielectric-barrier discharge ionization detector and a split injection unit with a split ratio of 1:20. Helium was chosen as the carrier gas. The column was heated to a constant temperature of 80˚C.

**Computational details:** The ORCA 6.0.^[1]^ computational software was used to carry out geometry optimization and the respective single point calculations. This work used the Becke 3-Parameter Lee-Yang-Parr (B3LYP)^[2]^ at the hybrid level of theory. The **def2-type** basis set of triple-zeta quality within the valence region, with an additional polarization function (**def2-TZVP**)^[3]^, was employed together with **Grimme’s D3BJ dispersion correction**, as implemented in the ORCA 6.0. software package to account for any longer-range interactions.^[4,5]^ The **effective core potentials (ECPs)** for molybdenum were likewise taken from the ORCA 6.0 implementation.^[6]^ All calculations were performed in the gas phase without the use of implicit solvation models. The visualization of the molecules **^1^[{Mo_3_Br}bpy]**, **^1^[{Mo_3_Br}phen]**, **^1^[{Mo_3_Br}dpbpy]**, **^1^[{Mo_3_Br}bphen]**, **^1^[{Mo_3_Br}fluo]**, **^1^[{Mo_3_Br}dppz]** as well as **^1^[{Mo_3_Br}dppzMe_2_]** of the respective LUMOs was performed using the Avogadro2 software package.^[7,8]^

**Stern-Volmer analysis:** For nanosecond transient emission spectroscopy, three sets of samples were prepared each containing 5 samples with 10 µM photosensitizer and varying concentration of catalyst (5, 10, 50, 100, 200 µM). The samples were prepared in a 1 cm quartz cuvette inside glovebox to maintain inert conditions using DMF as the solvent. Nanosecond transient emission spectroscopy measurements were obtained from the previously described set up.^[9]^ The pump pulse was obtained at the excitation (OPO used for excitation) of 470 nm by using a ND:YAG laser (Surelite) with a repetition rate of 10 Hz. The probe light source was a 75 W Xenon arc lamp which was captured and analysed using a Hamamatsu R928 photomultiplier. The pump and probe pulses were spatially overlapped at the sample position. For measurement, parameters were set same for all measurements (PMT = 400, shots and cycles = 10, Power = 120 µJ) The transient signals were obtained by using a detection system from Pascher instruments AB, Lund, Sweden. The collected data was processed using origin software and fitted using a single time constant in an exponential function.

# 2. Experimental Data

Commercially available reagents were purchased from Sigma Aldrich, TCI chemicals, BLDPharm and Fischer Scientific. The materials were used as obtained without further purification unless stated otherwise. (NH_4_)_2_[Mo_3_S_13_] x 2H_2_O,^[10]^ (TEA)_2_[Mo_3_S_7_Br_6_],^[11]^ dppz^[12]^ and dppzMe_2_^[13]^ were synthesized according to reported literature.

## 2.1. Synthesis of organic functionalized thiomolybdates

(TEA)_2_[Mo_3_S_7_Br_6_] (1 eq.) and the respective organic ligand (1 eq.) were dissolved or suspended in acetonitrile, heated to reflux and stirred overnight. The reaction mixture was filtered upon precipitation of the product. The product was washed with water, ethanol and ether and dried in a vacuum oven at 42°C overnight. Experimental details are shown in Table 1.

Table S1: Experimental details of the synthesis of all catalysts.

| **{Mo_3_Br}** | Ligand | Acetonitrile | Product | Yield |
| --- | --- | --- | --- | --- |
| 500 mg, 0.4 mmol, 1 eq | **bpy**, 62.5 mg, 0.4 mmol, 1 eq | 25 mL | **{Mo_3_Br}bpy** | 92% |
| 500 mg, 0.4 mmol, 1 eq | **phen**, 72.05 mg, 0.4 mmol, 1 eq | 25 mL | **{Mo_3_Br}phen** | 82% |
| 500 mg, 0.4 mmol, 1 eq | **dpbpy**, 121.9 mg, 0.4 mmol, 1 eq | 25 mL | **{Mo_3_Br}dpbpy** | 83% |
| 500 mg, 0.4 mmol, 1 eq | **bphen**, 132.9 mg, 0.4 mmol, 1 eq | 25 mL | **{Mo_3_Br}bphen** | 79% |
| 500 mg, 0.4 mmol, 1 eq | **fluo**, 72.8 mg, 0.4 mmol, 1 eq | 25 mL | **{Mo_3_Br}fluo** | 93% |
| 250 mg, 0.2 mmol, 1 eq | **dppz**, 56.4 mg, 0.4 mmol, 1 eq | 12 mL | **{Mo_3_Br}dppz** | 84% |
| 250 mg, 0.2 mmol, 1 eq | **dppzMe_2_**, 62.1 mg, 0.4 mmol, 1 eq | 12 mL | **{Mo_3_Br}dppzMe_2_** | 72% |

## 2.2. Photocatalytic HER Measurements

### **HER Catalysis**

Samples were prepared in an argon containing glovebox. Stock solutions of the catalyst (0.5 mg/mL) and the photosensitizer (1 mg/mL) were prepared in DMF. To a GC vial containing 220 mg ascorbic acid (2.5 M), 4.75 mL MeOH, catalyst solution (0.3 µM) and photosensitizer solution (20 µM) was added and filled with DMF to reach 5 mL solution. The vial was sealed and irradiated at λ = 467 nm (LED) for 6 h. Hydrogen was quantified with head-space GC.

Table S2: Catalytic TONs of all catalysts after 6 h, their mean value and standard deviation.

| TON {Mo_3_Br} | TON {Mo_3_Br}bpy | TON {Mo_3_Br}phen | TON {Mo_3_Br} dpbpy | TON {Mo_3_Br} bphen | TON {Mo_3_Br} fluo | TON {Mo_3_Br}dppz | TON {Mo_3_Br} dppzMe_2_ |
| --- | --- | --- | --- | --- | --- | --- | --- |
| 3595.89 | 3693.32 | 2897.61 | 3812.97 | 3764.99 | 4227.43 | 3310.18 | 2957.63 |
| 4041.70 | 3791.65 | 3296.72 | 3560.14 | 3314.78 | 3928.10 | 3353.05 | 3122.59 |
| 2858.63 | 3433.64 | 2970.80 | 3603.00 | 3636.64 | 3841.84 | 3511.28 | 2775.26 |
| 3599.87 | 3387.90 | 2626.74 | 3562.48 | 2820.00 | 3606.63 | 3375.60 | 2482.71 |
| 3914.81 | 3603.88 | 3594.40 | 3502.63 | 2633.48 | 3859.50 | 3290.15 | 2886.32 |
| 3967.55 | 3689.11 | 3254.60 | 3911.42 | 2736.38 | 3281.42 | 3198.19 | 2902.96 |
| 3770.14 | 3752.32 | 3762.08 | 3779.15 | 3739.34 | 3639.20 | 3192.73 | 3022.14 |
| 3685.10 | 3246.93 | 3358.40 | 3520.10 | 3679.28 | 4245.68 | 3013.10 | 3193.20 |
| 3945.37 | 3228.48 | 3178.49 | 3507.12 | 3917.44 | 4338.43 | 3664.05 | 3126.70 |
| 3870.26 | 2912.76 | 3066.27 | 3062.25 | 4114.91 | 4140.12 | 3075.67 | 2663.13 |
| 3656.32 | 2957.61 | 3155.50 | 3131.19 | 3539.41 | 3664.05 | 3277.73 | 3305.97 |
| 4137.00 | 3067.49 | 3282.45 | 3341.33 | 3820.96 | 4116.55 | 3209.17 | 2987.48 |
| 4138.14 | 3174.95 | 3393.40 | 3463.73 | 3389.51 | 4054.79 | - | 3202.76 |
| 3858.13 | 3517.90 | 3288.66 | 3830.04 | - | 3878.82 | - | - |
| average value | | | | | | | |
| 3788.49 | 3389.03 | 3223.29 | 3541.97 | 3469.78 | 3915.90 | 3289.24 | 2971.45 |
| standard deviation | | | | | | | |
| 323.18 | 298.70 | 283.70 | 248.40 | 417.36 | 296.46 | 177.81 | 232.54 |

### **Catalytic reference measurements without photosensitizer**

Reference measurements were performed in triplicates under comparable conditions only without the addition of [Ru(bpy)_3_]^2+^.

Table S3: Catalytic TONs of all catalysts in absence of [Ru(bpy)_3_]^2+^ after 6 h.

| Catalyst | TON |
| --- | --- |
| **{Mo_3_Br}** | 35.63 ± 8.95 |
| **{Mo_3_Br}bpy** | 38.30 ± 10.82 |
| **{Mo_3_Br}phen** | 49.71 ± 5.99 |
| **{Mo_3_Br}dpbpy** | 42.73 ± 15.67 |
| **{Mo_3_Br}bphen** | 32.96 ± 14.63 |
| **{Mo_3_Br}fluo** | 30.46 ± 4.94 |
| **{Mo_3_Br}dppz** | 33.69 ± 9.59 |
| **{Mo_3_Br}dppzMe_2_** | 34.85 ± 3.08 |
|  |  |

### **Determination of photonic efficiency**

To determine the photonic efficiency (PE) the power fluence of the LED at the sample was determined using a Thorlabs PM400 Optical Power Meter. The sensor was placed in the position and at a height comparable to the bottom of the sample vials. The PE is determined using the photon fluence rate *q* which is calculated based on the experimentally determined power *P* of the LED (15 ± 1 mW), the emission wavelength $\lambda$ = 467 nm, the Planck constant *h*, the speed of light *c* and the Avogadro constant *N_A_*.

$q=\frac{P\cdot\lambda}{h\cdot c\cdot N_{A}}$	[1]

The total number of photons $n_{\text{ph}}$ can be calculated by multiplying the photon fluence rate *q* with the time *t* and the area *A*_Vial_ of the vial (= 1.227 cm^2^).

$n_{\text{ph}}=q\cdot t\text{ }\text{⋅}A_{Vial}\text{ }$	[2]

The PE is calculated using equation [3], were $n_{\text{H}_{\text{2}}\text{,abs}}$ is the amount of hydrogen produced during the catalysis.^[14,15]^

$\text{PE}=\frac{n_{\text{H}_{\text{2}}\text{,abs}}}{n_{\text{ph}}}$	[3]

TableS4: PE calculated based on averaged TONs.

| Catalyst | Photonic efficiency / 10^-3^ |
| --- | --- |
| **{Mo_3_Br}** | 3.66 ± 0.49 |
| **{Mo_3_Br}bpy** | 3.28 ± 0.44 |
| **{Mo_3_Br}phen** | 3.12 ± 0.42 |
| **{Mo_3_Br}dpbpy** | 3.42 ± 0.41 |
| **{Mo_3_Br}bphen** | 3.35 ± 0.62 |
| **{Mo_3_Br}fluo** | 3.78 ± 0.47 |
| **{Mo_3_Br}dppz** | 3.18 ± 0.33 |
| **{Mo_3_Br}dppzMe_2_** | 2.87 ± 0.36 |

## 2.3. Characterization of compounds

### **^1^H-NMR spectra**

Figure SS1: ^1^H-NMR spectra of **{Mo_3_Br}bpy** (blue) and bpy (red) in DMSO-d_6_.

Figure S2: ^1^H-NMR spectra of **{Mo_3_Br}phen** (blue) and phen (red) in DMSO-d_6_.

Figure S3: ^1^H-NMR spectra of **{Mo_3_Br}dpbpy** (blue) and dpbpy (red) in DMSO-d_6_.

Figure S4: ^1^H-NMR spectra of **{Mo_3_Br}bphen** (blue) and bphen (red) in DMSO-d_6_.

Figure S5: ^1^H-NMR spectra of **{Mo_3_Br}fluo** (blue) and fluo (red) in DMSO-d_6_.

Figure S6: ^1^H-NMR spectra of **{Mo_3_Br}dppz** (blue) and dppz (red) in DMSO-d_6_.

Figure S7: ^1^H-NMR spectra of **{Mo_3_Br}dppzMe_2_** (blue) and dppzMe_2_ (red) in DMSO-d_6_.

### **CHN Elemental Analysis**

**{Mo_3_Br}bpy**:

Elemental analysis for Mo_3_S_7_Br_5_C_18_N_3_H_28_ in wt-% (calculated): C 16.05 (18.04), H 1.76 (2.36), N 3.26 (3.51), S 20.52 (18.73)

**{Mo_3_Br}phen**:

Elemental analysis for Mo_3_S_7_Br_5_C_20_N_3_H_28_ in wt-% (calculated): C 18.51 (19.65), H 2.08 (2.31), N 3.38 (3.44), S 19.25 (18.36)

**{Mo_3_Br}dpbpy**:

Elemental analysis for Mo_3_S_7_Br_5_C_30_N_3_H_36_ in wt-% (calculated): C 29.44 (26.68), H 2.39 (2.69), N 3.39 (3.11), S 16.19 (16.62)

**{Mo_3_Br}bphen**:

Elemental analysis for Mo_3_S_7_Br_5_C_32_N_3_H_36_ in wt-% (calculated): C 27.84 (27.96), H 2.71 (2.64), N 3.14 (3.06), S 16.67 (16.33)

**{Mo_3_Br}fluo**:

Elemental analysis for Mo_3_S_7_Br_5_C_19_ON_3_H_26_ in wt-% (calculated): C 18.19 (18.64), H 3.30 (2.14), N 3.02 (3.43), S 17.40 (18.33)

**{Mo_3_Br}dppz**:

Elemental analysis for Mo_3_S_7_Br_5_C_26_N_5_H_30_ in wt-% (calculated): C 24.72 (23.58), H 2.90 (2.28), N 5.27 (5.29), S 16.14 (16.95)

**{Mo_3_Br}dppzMe_2_**:

Elemental analysis for Mo_3_S_7_Br_5_C_28_N_5_H_34_ in wt-% (calculated): C 22.91 (24.87), H 1.89 (2.53), N 4.93 (5.18), S 17.48 (16.59)

### **ATR-FT-IR spectra**

Figure S8: ATR-FT-IR spectrum of **{Mo_3_Br}bpy**.

Figure S9: ATR-FT-IR spectrum of **{Mo_3_Br}phen**.

Figure S10: ATR-FT-IR spectrum of **{Mo_3_Br}dpbpy**.

Figure S11: ATR-FT-IR spectrum of **{Mo_3_Br}bphen**.

Figure S12: ATR-FT-IR spectrum of **{Mo_3_Br}fluo**.

Figure S13: ATR-FT-IR spectrum of **{Mo_3_Br}dppz**.

Figure S14: ATR-FT-IR spectrum of **{Mo_3_Br}dppzMe_2_**.

### **UV-Vis spectra**

Figure S15: UV-Vis spectrum of **{Mo_3_Br}bpy**, 0.05 mM in DMF.

Figure S16: UV-Vis spectrum of **{Mo_3_Br}phen**, 0.05 mM in DMF.

Figure S17: UV-Vis spectrum of **{Mo_3_Br}dpbpy**, 0.05 mM in DMF.

Figure S18: UV-Vis spectrum of **{Mo_3_Br}bphen**, 0.05 mM in DMF.

Figure S19: UV-Vis spectrum of **{Mo_3_Br}fluo**, 0.05 mM in DMF.

Figure S20: UV-Vis spectrum of **{Mo_3_Br}dppz**, 0.05 mM in DMF.

Figure S21: UV-Vis spectrum of **{Mo_3_Br}dppzMe_2_**, 0.05 mM in DMF.

### **Single crystal XRD data**

The crystal structure of **{Mo_3_Br}bpy** was previously reported by Nandi and collegues.^[16]^ The authors collected independent SC-XRD data to verify the structure of the compound used.

Table S5: Crystal data and structure refinement for **{Mo_3_Br}bpy**.

| Deposition Number | 874761 |
| --- | --- |
| Empirical formula | C_20_H_31_N_4_S_7_Mo_3_Br_5_ |
| Formula weight | 1239.270 |
| Temperature/K | 120 |
| Crystal system | triclinic |
| Space group | P-1 |
| a/Å | 11.4632(5) |
| b/Å | 12.8371(6) |
| c/Å | 13.0923(6) |
| α/° | 79.760(4) |
| β/° | 86.858(4) |
| γ/° | 66.292(3) |
| Volume/Å^3^ | 1735.60(14) |
| Z | 2 |
| ρ_calc_g/cm^3^ | 2.371 |
| μ/mm^‑1^ | 7.265 |
| F(000) | 1174.0 |
| Crystal size/mm^3^ | 0.45 × 0.19 × 0.052 |
| Radiation | Mo Kα (λ = 0.71073) |
| 2Θ range for data collection/° | 4.82 to 56.12 |
| Index ranges | -15 ≤ h ≤ 15, -16 ≤ k ≤ 16, -17 ≤ l ≤ 17 |
| Reflections collected | 21993 |
| Independent reflections | 8268 [R_int_ = 0.0219, R_sigma_ = 0.0211] |
| Data/restraints/parameters | 8268/0/357 |
| Goodness-of-fit on F^2^ | 1.223 |
| Final R indexes [I>=2σ (I)] | R_1_ = 0.0315, wR_2_ = 0.0648 |
| Final R indexes [all data] | R_1_ = 0.0412, wR_2_ = 0.0713 |
| Largest diff. peak/hole / e Å^-3^ | 1.02/-0.97 |

Figure S22: ORTEP representation of the crystal structure of **{Mo_3_Br}bpy**, C (grey), H (white), Br (red), Mo (dark blue), N (blue), S (yellow), solvent molecules were removed for clarity, probability ellipsoids at 50%.

Table S6: Crystal data and structure refinement for **{Mo_3_Br}bphen**.

| Deposition Number | 2530461 |
| --- | --- |
| Empirical formula | Mo_3_S_7_Br_5_C_38_H_50_N_5_O_2_ |
| Formula weight | 1520.627 |
| Temperature/K | 120 |
| Crystal system | orthorhombic |
| Space group | Pbca |
| a/Å | 14.1377(2) |
| b/Å | 29.3971(6) |
| c/Å | 24.0351(6) |
| α/° | 90 |
| β/° | 90 |
| γ/° | 90 |
| Volume/Å^3^ | 9989.2(4) |
| Z | 8 |
| ρ_calc_g/cm^3^ | 2.022 |
| μ/mm^‑1^ | 5.073 |
| F(000) | 5896.6 |
| Crystal size/mm^3^ | 0.31 × 0.08 × 0.07 |
| Radiation | Mo Kα (λ = 0.71073) |
| 2Θ range for data collection/° | 5.92 to 55.96 |
| Index ranges | -18 ≤ h ≤ 16, -38 ≤ k ≤ 33, -27 ≤ l ≤ 31 |
| Reflections collected | 28926 |
| Independent reflections | 11899 [R_int_ = 0.0584, R_sigma_ = 0.0626] |
| Data/restraints/parameters | 11899/0/549 |
| Goodness-of-fit on F^2^ | 1.169 |
| Final R indexes [I>=2σ (I)] | R_1_ = 0.0608, wR_2_ = 0.1305 |
| Final R indexes [all data] | R_1_ = 0.1011, wR_2_ = 0.1613 |
| Largest diff. peak/hole / e Å^-3^ | 1.74/-2.10 |
|  |  |

Figure S23: ORTEP representation of the crystal structure of **{Mo_3_Br}bphen**, C (grey), H (white), Br (red), Mo (dark blue), N (blue), S (yellow), solvent molecules were removed for clarity, probability ellipsoids at 50%.

## 2.4. Computational Data

Table S7: Comparison of TONs, reductive onset potential and calculated ligand-centered π(L)/π*(L) and metal-centered d(CAT)/n(CAT) orbitals. The d(CAT) orbitals represent the HOMO of the compounds and π*(L) represents the LUMO of the compound, except for **{Mo_3_Br}** where the LUMO is the n(CAT) orbital.

| **Catalyst** | **HER TONs** | E_onset_ / V | π (L) / eV | d(CAT) / eV | π *(L) / eV | n(CAT) / eV |
| --- | --- | --- | --- | --- | --- | --- |
| **{Mo_3_Br}fluo** | **3916** | -1.44 | -7.6055 | -6.3594 | -3.4704 | -2.9488 |
| **{Mo_3_Br}** | **3788** | -1.35 | / | -5.9134 | / | -2.5690 |
| **{Mo_3_Br}dpbpy** | **3542** | -0.97 | -8.0305 | -5.5129 | -3.7000 | -2.7320 |
| **Mo_3_Br}bphen** | **3470** | -0.99 | -7.6403 | -5.4968 | -3.6221 | -2.7167 |
| **{Mo_3_Br}bpy** | **3389** | -0.92 | -8.5495 | -5.6658 | -3.8873 | -2.8992 |
| **{Mo_3_Br}dppz** | **3289** | -1.01 | -7.5407 | -5.3314 | -4.2302 | -3.1418 |
| **{Mo_3_Br}phen** | **3223** | -0.95 | -8.4206 | -5.6387 | -3.8319 | -2.6894 |
| **{Mo_3_Br}dppzMe_2_** | **2971** | -1.00 | -7.5649 | -5.5461 | -3.7997 | -2.7756 |

### **Visualization of frontier orbitals of all catalysts**

Table S 8: Visualization of ligand-centered π(L)/π*(L) and metal-centered n(CAT)/d(CAT) orbitals of {Mo₃Br} and ligand-substituted derivatives.

|  | Occupied | | Virtuelle | |
| --- | --- | --- | --- | --- |
|  | π(L) | n(CAT) | π(L)* | d(CAT) |
| **{Mo_3_Br}** | - |  |  |  |
|  |  | HOMO |  | LUMO |
| **{Mo3Br}fluo** |  |  |  |  |
|  | HOMO-9 | HOMO | LUMO | LUMO+2 |
| **{Mo_3_Br}bpy** |  |  |  |  |
|  | HOMO-18 | HOMO | LUMO | LUMO+2 |
| **{Mo_3_Br}dpbpy** |  |  |  |  |
|  | HOMO-17 | HOMO | LUMO | LUMO+3 |
| **{Mo_3_Br}phen** |  |  |  |  |
|  | HOMO-18 | HOMO | LUMO | LUMO+2 |
| **{Mo_3_Br}bphen** |  |  |  |  |
|  | HOMO-14 | HOMO | LUMO | LUMO+2 |
| **{Mo_3_Br}dppz** |  |  |  |  |
|  | HOMO-14 | HOMO | LUMO | LUMO+3 |
| **{Mo_3_Br}dppzMe_2_** |  |  |  |  |
|  | HOMO-12 | HOMO | LUMO | LUMO+3 |

## 2.5 Catalyst stability

In order to assess the stability of the catalysts under irradiation, catalyst solutions (10 mM) in DMSO-d_6_ were irradiated for 6 h using a 467 nm LED and investigated using ^1^H-NMR spectroscopy before and after irradiation. Due to the low solubility of **{Mo_3_Br}phen** and **{Mo_3_Br}dppzMe_2_** the resulting suspensions were filtrated prior to irradiation.

Figure S24: ^1^H-NMR spectra of **{Mo_3_Br}bpy** before (blue) and after (red) irradiation (6 h, 467 nm) in DMSO-d_6_.

Figure S25: ^1^H-NMR spectra of **{Mo_3_Br}phen** before (blue) and after (red) irradiation (6 h, 467 nm) in DMSO-d_6_.

Figure S26: ^1^H-NMR spectra of **{Mo_3_Br}dpbpy** before (blue) and after (red) irradiation (6 h, 467 nm) in DMSO-d_6_.

Figure S27: ^1^H-NMR spectra of **{Mo_3_Br}bphen** before (blue) and after (red) irradiation (6 h, 467 nm) in DMSO-d_6_.

Figure S28: ^1^H-NMR spectra of **{Mo_3_Br}fluo** before (blue) and after (red) irradiation (6 h, 467 nm) in DMSO-d_6_.

Figure S29: ^1^H-NMR spectra of **{Mo_3_Br}dppz** before (blue) and after (red) irradiation (6 h, 467 nm) in DMSO-d_6_.

Figure S30: ^1^H-NMR spectra of **{Mo_3_Br}dppzMe_2_** before (blue) and after (red) irradiation (6 h, 467 nm) in DMSO-d_6_.

# 3. References

[1]	F. Neese, “Software Update: The ORCA Program System—Version 6.0” *Wiley Interdisciplinary Reviews: Computational Molecular Science* **15**, (2025): e70019.

[2]	A. D. Becke, “Density‐functional thermochemistry. III. The role of exact exchange” *The Journal of Chemical Physics* **98**, (1993): 5648–5652.

[3]	F. Weigend, R. Ahlrichs, “Balanced basis sets of split valence, triple zeta valence and quadruple zeta valence quality for H to Rn: Design and assessment of accuracy” *Physical Chemistry Chemical Physics* **7**,(2005): 3297–3305.

[4]	S. Grimme, S. Ehrlich, L. Goerigk, “Effect of the damping function in dispersion corrected density functional theory” *Journal of Computational Chemistry* **32**, (2011): 1456–1465.

[5]	S. Grimme, J. Antony, S. Ehrlich, H. Krieg, “A consistent and accurate ab initio parametrization of density functional dispersion correction (DFT-D) for the 94 elements H-Pu” *Journal of Chemical Physics* **132**, (2010): 154104.

[6]	D. Andrae, U. Häußermann, M. Dolg, H. Stoll, H. Preuß, “Energy-adjusted ab initio pseudopotentials for the second and third row transition elements” *Theoretica chimica acta* **77**, (1990): 123–141.

[7]	M. D. Hanwell, D. E. Curtis, D. C. Lonie, T. Vandermeerschd, E. Zurek, G. R. Hutchison, “Avogadro: An advanced semantic chemical editor, visualization, and analysis platform” *Journal of Cheminformatics* **4**, (2012): 17.

[8]	Avogadro: an open-source molecular builder and visualization tool. Version 1.102.3. http://avogadro.cc/.

[9]	M. Stephenson, C. Reichardt, M. Pinto, M. Wächtler, T. Sainuddin, G. Shi, H. Yin, S. Monro, E. Sampson, B. Dietzek, S. A. McFarland, “Ru(II) dyads derived from 2-(1-pyrenyl)-1H-imidazo[4,5-f][1,10]phenanthroline: Versatile photosensitizers for photodynamic applications” *Journal of Physical Chemistry A* **118**, (2014): 10507–10521.

[10]	M. Dave, A. Rajagopal, M. Damm-Ruttensperger, B. Schwarz, F. Nägele, L. Daccache, D. Fantauzzi, T. Jacob, C. Streb, “Understanding homogeneous hydrogen evolution reactivity and deactivation pathways of molecular molybdenum sulfide catalysts” *Sustainable Energy Fuels* **2**, (2018): 1020–1026.

[11]	V. P. Fedin, M. N. Sokolov, Y. V Mironov, B. A. Kolesov, S. V Tkachev, V. Y. Fedorov, “Triangular Thiocomplexes of Molybdenum: Reactions with Halogens, Hydrohalogen Acids and Phosphines” *Inorganica Chimica Acta* **167**, (1990): 39–45.

[12]	W. Travis, C. E. Knapp, C. N. Savory, A. M. Ganose, P. Kafourou, X. Song, Z. Sharif, J. K. Cockcroft, D. O. Scanlon, H. Bronstein, R. G. Palgrave, “Hybrid Organic-Inorganic Coordination Complexes as Tunable Optical Response Materials” *Inorganic Chemistry* **55**, (2016): 3393–3400.

[13]	A. Kleineweischede, J. Mattay, “Synthesis of Amino- and Bis(bromomethyl)-Substitued Bi- and TetradentateN-Heteroaromatic Ligands: Building Blocks for Pyrazino-Functionalized Fullerene Dyads” *European Journal of Organic Chemistry* **2006**, (2006) 947–957.

[14]	E. F. Schuberiil, *Light Emitting Diodes*, 3rd Edition *E. Fred Schubert,* **2018**.

[15] S. H. Kolbinger, L. Haxha, P. C. Schröder, D. Kowalczyk, L. Senz, L. Schleicher, A. Mukherjee, D. Malcolm, C. L. Kufner, D. Ziegenbalg, C. Streb, “Fewer photons, more hydrogen: effects of dynamic irradiation on light-driven hydrogen evolution by thiomolybdate catalysts” *Sustain. Energy Fuels* **10**, (2026): 1313–1320.

[16]	G. Nandi, S. Sarkar, B. S. Reddy, T. Y. Kim, K. M. Tripathi, “Heteroleptic bipyridine complex: Synthesis, spectral and structural analyses, and interconversion of its {Mo_3_S_7_} core to {Mo_3_S_4_} core” *Journal of Molecular Structure* **1234**, (2021): 130138.
